# Spontaneous Expulsive Suprachoroidal Hemorrhage in a Middle-Aged Patient With Hypertension, Type II Diabetes, and Associated Retinopathy

**DOI:** 10.7759/cureus.58579

**Published:** 2024-04-19

**Authors:** Koki Honzawa, Hiroshi Horiguchi, Masaki Nakamura, Satoshi Katagiri, Hisato Gunji, Tadashi Nakano

**Affiliations:** 1 Department of Ophthalmology, The Jikei University School of Medicine, Tokyo, JPN

**Keywords:** diabetic retinopathy, type ii diabetes, spontaneous expulsive suprachoroidal hemorrhage, hypertension, angle closure

## Abstract

We report the clinical course of spontaneous expulsive suprachoroidal hemorrhage (SESCH) in a middle-aged man. A 50-year-old man with a history of uncontrolled hypertension and type II diabetes presented with massive preretinal hemorrhage in the posterior pole of the right eye (RE). Two weeks later, he presented with elevated intraocular pressure (IOP) and a nearly obliterated anterior chamber with coagulated blood behind the lens in the RE. We performed two rounds of surgery, including cataract surgery, vitrectomy, and sclerotomy. The choroidal detachment was clearly visible behind the posterior capsule during the cataract surgery. The surgical intervention successfully lowered the IOP and alleviated the pain. In rare cases of SESCH, maintaining awareness when patients show vulnerability in their choroidal vessels is of high importance.

## Introduction

Suprachoroidal hemorrhage (SESCH) is a vision-threatening disease characterized by the accumulation of hemorrhage between the choroid and sclera due to rupture of the long or short ciliary arteries. An expulsive hemorrhage is marked by substantial subretinal and vitreous hemorrhage caused by bleeding from the short posterior ciliary artery during surgery. SESCH is commonly triggered by surgical procedures or traumatic events, whereas spontaneous expulsive SESCH is a rare disease. Based on the PubMed database (accessed on 20th March 2024), only 60 eyes of 55 patients with SESCH have been reported [1-9]. Thus, further case accumulation is needed to understand the disease.

In this report, we present the clinical course of a rare case of SESCH in a middle-aged man with a low risk of ocular disease that could cause SESCH.

## Case presentation

**Figure 1:**
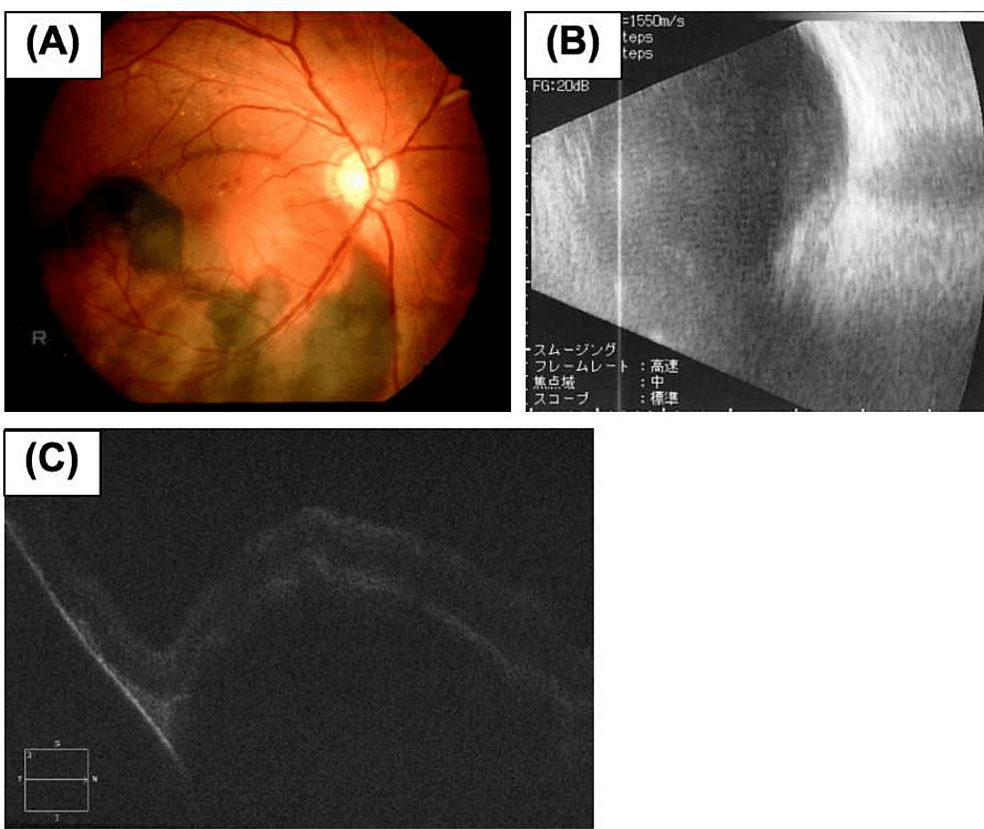
Fundus examination images of the right eye before the onset of spontaneous expulsive suprachoroidal hemorrhage. (A) The fundus photograph of the right eye at the first examination at a nearby clinic revealed a massive subretinal hemorrhage in the inferior region of the posterior pole, including the macula. (B and C) Examination during the first visit to our hospital. At that time, a vitreous hemorrhage occurred in the right eye. (B) B-scan ultrasonography showed vitreous opacity and a mass with high internal reflectivity. (C) Optical coherence tomography image, which is of poor quality due to vitreous hemorrhage, revealed a massive hyporeflective lesion between the retina and the underlying retinal pigment epithelium.

A 50-year-old man visited a clinic with metamorphopsia in his right eye (RE), massive subretinal hemorrhage affecting the macula in the RE, and pre-proliferative diabetic retinopathy (DR) and hypertensive retinopathy in both eyes (Figure 1A). He had a history of type II diabetes and hypertension for two years but had missed clinic appointments for one year. He had no other medical conditions, prior surgeries, or anticoagulant use. Following the clinic visit, physicians re-diagnosed uncontrolled hypertension (blood pressure 228/124 mmHg) and type II diabetes (HbA1c 9.0) and immediately initiated medication. Blood investigations including coagulation proﬁle were within normal limits. One week later (Day 1), he was referred to our hospital with visual acuity of hand motion and 20/17 in the RE and left eye (LE), respectively, with normal intraocular pressure (IOP). Fundoscopy showed fundus-obscuring vitreous and subretinal hemorrhages in the RE. B-scan ultrasonography and optical coherence tomography (OCT) showed a massive subretinal hemorrhage in the RE (Figures 1B, 1C). Fluorescein angiography of the LE showed multiple retinal microaneurysms and non-perfused areas. We scheduled vitreous surgery for vitreous hemorrhage of the RE and panretinal photocoagulation for pre-proliferative DR of the LE.

**Figure 2:**
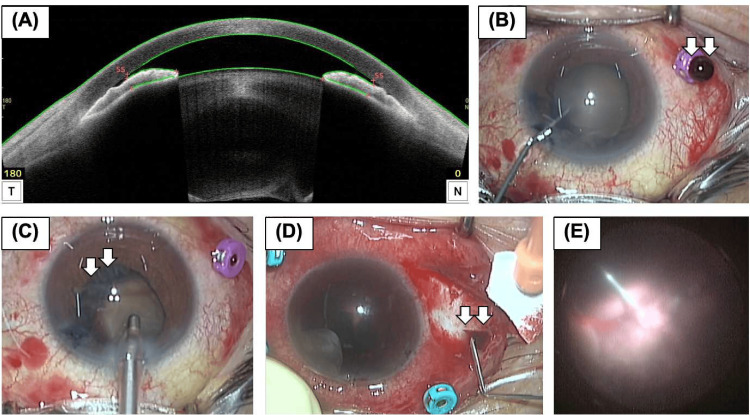
Preoperative and intraoperative findings of the right eye. (A) Preoperative anterior segment optical coherence tomography revealed an extreme forward displacement of the lens-iris septum. (B and C) Intraoperative findings of the first surgery. (B) A copious and thick flux of blood immediately flowed out of the cannula (white arrow). (C) The choroidal detachment was visible behind the lens capsule during irrigation and aspiration (white arrow). (D and E) Intraoperative findings of the second surgery. (D) The suprachoroidal hemorrhage was drained (white arrow) from the scleral incision on the temporal side. (E) The fundus image revealed choroidal detachment and a white-colored retina in the posterior and peripheral areas.

On day 7, he developed sudden pain. IOP was elevated up to 70 mmHg in the RE, and slit-lamp examination showed an edematous cornea, a nearly obliterated anterior chamber, and coagulated blood behind the lens. Anterior segment OCT revealed forward displacement of the lens-iris septum (Figure 2A). We suspected acute angle-closure glaucoma due to increased vitreous hemorrhage. On day 8, we performed phacoemulsification without intraocular lens implantation. A single 27-gauge vitrectomy cannula was placed to reduce intravitreal pressure and maintain the depth of the anterior chamber. Copious blood flowed from the cannula (Figure 2B), which was drained as much as possible. Intraoperative findings of iris stroma prolapse toward side-port incisions, choroidal detachment behind the lens capsule, and rupture of Zinn’s zonule, led to the diagnosis of SESCH. On day 9, he was referred for the resolution of ocular pain with a reduction in IOP to 34 mmHg. Slit-lamp examination revealed a hyphema that kept the fundus invisible. He was treated postoperatively with topical steroids, 1% atropine sulfate hydrate, antibiotics, systemic steroids, and 20% mannitol intravenously. On day 17, the IOP increased again to 60 mmHg, and he experienced a flare-up of ocular pain. On day 23, we performed anterior chamber irrigation, 25-gauge vitrectomy via the pars plana, and sclerotomy to reduce ocular pain due to the elevated IOP (Figure 2C). Scleral incisions were made and some SESCH was drained using compression (Figure 2D). Anterior chamber irrigation and vitrectomy reduced bleeding in the anterior chamber and vitreous cavity. Intraoperative examination revealed choroidal detachment and a white-colored retina, suggesting ischemic changes in the posterior and peripheral areas (Figure 2E). At the end of surgery, the anterior chamber was filled with blood. On day 24, the ocular pain disappeared, and the IOP decreased to 4 mmHg. However, visual acuity had no light perception with hyphema, and fundus examination was impossible. The low IOP, hyphema, and absence of ocular pain persisted until the last visit to our hospital (five months after the second surgery).

## Discussion

Reportedly, various systemic and ocular conditions are risk factors for SESCH [1]. Systemic conditions include advanced age (>60 years), hypertension, diabetes mellitus, anemia, thrombocytopenia, cardiovascular diseases, and associated medications for anticoagulation therapy and Valsalva maneuvers [1]. Ocular conditions include age-related macular degeneration, glaucoma, pseudophakia, and longer axial length [1]. Our patient had unmedicated hypertension, type II diabetes, and diabetic retinopathy with subretinal and vitreous hemorrhages. Among the reported 60 eyes of 55 patients with SESCH, 14 eyes of 14 patients (23.3%) had some systemic risk factors with no ocular abnormalities. Most patients had thrombocytopenia or coagulation abnormalities owing to systemic disease and/or anticoagulation therapy. Additionally, only one patient under the age of 60 without systemic risk factors of coagulation abnormalities or a history of Valsalva maneuvers developed SESCH [2]. Although the pathogenesis of SESCH remains unclear, studies suggested factors like inflammatory necrosis, sudden compression and decompression, and infection of the choroidal vessels. We hypothesized that our patient had some vulnerability in the choroidal vessels due to poorly controlled hypertension and diabetes mellitus, which led to the sudden onset of SESCH. Additionally, diabetic retinopathy primarily affects retinal vasculature and tissues [10], which may be less associated with SESCH occurrence.

The diagnosis of SESCH is usually based on fundus examination, OCT, and ultrasonography before ophthalmic treatment. In contrast, we suspected SESCH during the first surgery because of the significant outflow of SESCH upon cannula insertion and the presence of choroidal detachment behind the lens capsule during cataract surgery. Vitreous hemorrhage alone could induce angle closure [11], although this is extremely rare. Therefore, it is vital to evaluate the posterior pushing mechanism of angle closure using ultrasonography, particularly when there is a possibility of underlying posterior ocular diseases. This evaluation would have allowed for the appropriate choroidal drainage procedure during the patient’s first surgery.

On the timing of surgical intervention, perspectives have not yet been unified. A prior study suggested that a seven-day interval could be the best time for clot lysis to occur, which would enable effective posterior sclerotomy for drainage [12]. However, waiting for surgical intervention for hemorrhage may result in progression to retinal detachment or lead to chronic atrophy, potentially resulting in blindness [13]. Surgical drainage is indicated for progressive angle closure, appositional CD, and elevated IOP [14,15]. Our patient had angle closure and elevated IOP which did not respond to drug therapy, so he required urgent surgery.

## Conclusions

We presented a rare case of SESCH with systemic diseases such as poorly controlled hypertension, type II diabetes, and ocular diseases of diabetic and hypertensive retinopathy associated with subretinal and vitreous hemorrhages before the onset of SESCH. SESCH is a severe ocular emergency, which leads to a rapid and profound decrease in vision due to elevated IOP and displacement of intraocular structures. Despite its rarity, it is essential to maintain awareness of SESCH in patients showing choroidal vessel vulnerability due to systemic and ocular factors.
